# *POLE* somatic mutations in advanced colorectal cancer

**DOI:** 10.1002/cam4.1245

**Published:** 2017-10-26

**Authors:** Joana Guerra, Carla Pinto, Diana Pinto, Manuela Pinheiro, Romina Silva, Ana Peixoto, Patrícia Rocha, Isabel Veiga, Catarina Santos, Rui Santos, Verónica Cabreira, Paula Lopes, Rui Henrique, Manuel R. Teixeira

**Affiliations:** ^1^ Department of Genetics Portuguese Oncology Institute of Porto (IPO‐Porto) Porto Portugal; ^2^ Department of Pathology Portuguese Oncology Institute of Porto (IPO‐Porto) Porto Portugal; ^3^ Department of Pathology and Molecular Immunology Institute of Biomedical Sciences Abel Salazar (ICBAS) University of Porto Porto Portugal

**Keywords:** Colorectal cancer, non‐MSI‐H phenotype, oncogene mutation, *POLE* mutations

## Abstract

Despite all the knowledge already gathered, the picture of somatic genetic changes in colorectal tumorigenesis is far from complete. Recently, germline and somatic mutations in the exonuclease domain of polymerase epsilon, catalytic subunit (*POLE*) gene have been reported in a small subset of microsatellite‐stable and hypermutated colorectal carcinomas (CRCs), affecting the proofreading activity of the enzyme and leading to misincorporation of bases during DNA replication. To evaluate the role of *POLE* mutations in colorectal carcinogenesis, namely in advanced CRC, we searched for somatic mutations by Sanger sequencing in tumor DNA samples from 307 cases. Microsatellite instability and mutation analyses of a panel of oncogenes were performed in the tumors harboring *POLE* mutations. Three heterozygous mutations were found in two tumors, the c.857C>G, p.Pro286Arg, the c.901G>A, p.Asp301Asn, and the c.1376C>T, p.Ser459Phe. Of the *POLE*‐mutated CRCs, one tumor was microsatellite‐stable and the other had low microsatellite instability, whereas *KRAS* and *PIK3CA* mutations were found in one tumor each. We conclude that *POLE* somatic mutations exist but are rare in advanced CRC, with further larger studies being necessary to evaluate its biological and clinical implications.

## Introduction

Colorectal cancer (CRC) is one of the major causes of morbidity and mortality throughout the world and accounts for over 9% of all cancer cases diagnosed in 2012 1. The etiological factors and pathogenic mechanisms underlying CRC development appear to be complex and heterogeneous. The majority of CRC cases occur sporadically, arising through the sequential accumulation of multiple genetic and/or epigenetic alterations involving genes that regulate cell growth and differentiation 2, 3. Several crucial gene defects in sporadic CRC have been identified, and several molecular pathways have been described, such as the chromosomal instability (CIN), the microsatellite instability (MSI), and the CpG island methylator phenotype (CIMP) pathways 4. These pathways are not mutually exclusive as a tumor can occasionally show features of multiple pathways, although the extent and nature of this overlap remains to be determined 2, 4.

Despite the enormous progress in defining some of the common genetic and epigenetic alterations, the picture of somatic genetic changes in colorectal tumorigenesis is far from complete. To fill in the gaps, The Cancer Genome Atlas (TCGA) exome sequencing project published the result of full genomic profiling of 224 CRC samples 5. This work confirmed many previous mutational findings in CRC and additionally identified new rare findings, such as mutations in the exonuclease domain of polymerase epsilon, catalytic subunit (*POLE*) gene in approximately 3% of sporadic microsatellite‐stable (MSS) but hypermutated CRCs 5, 6, 7. The *POLE* gene is located in 12q24.33 and encodes the proofreading (exonuclease) subunit of polymerase epsilon (POLE) with 2286 amino acids. Palles and collaborators, in 2013, found a subset of patients with MSS CRC that harbor germline *POLE* exonuclease domain mutations (EDMs) 6. Subsequently, Stenzinger and coworkers detected somatic *POLE* EDMs in 12.3% of MSS sporadic CRC 8. Additionally, *POLE* EDMs were found in about 7% of sporadic endometrial cancer (EC) and, similarly to what was observed in CRC, were associated with an ultramutator and MSS phenotype 7, 9. POLE has an essential role in chromosomal DNA replication, namely in the leading‐strand synthesis and recognition and removal of mispaired nucleotides by its proofreading capacity through the POLE exonuclease domain, which is crucial for the maintenance of replication fidelity 10, 11, 12. The aim of this study was to evaluate the frequency and role of *POLE* somatic mutations in colorectal carcinogenesis, namely in advanced CRC.

## Material and Methods

### Tumor samples

This study includes 307 patients with CRC for whom *KRAS* exon 2 mutations had been requested to the Genetics Department of the Portuguese Oncology Institute of Porto (IPO‐Porto) between August 2008 and December 2012 and from whom there was sufficient tumor DNA/sample left for further analyses.

### DNA extraction

DNA was isolated from tumor areas containing at least 50% of tumor cells, delimited by a pathologist, using the QIAamp^®^ DNA FFPE Tissue Kit (Qiagen, Hilden, Germany) and following the manufacturer's instructions. In samples with mutations, the normal mucosa was also extracted from the same slide, to confirm the somatic status of mutations, using the same approach.

### *POLE* mutation analysis

Somatic mutation screening of the exonuclease domain of *POLE* (exons 9–14) was performed by Sanger sequencing. Primer sequences were kindly provided by Professor Ian Tomlinson from the Wellcome Trust Centre for Human Genetics, University of Oxford, United Kingdom. Sanger sequencing was performed using the BigDye^®^ Terminator v1.1 or v3.1 Cycle Sequencing Kit (Applied Biosystems, Foster City, CA, USA), following the manufacturer's instructions, and the products were analyzed on an ABI PRISM^™^ 310 Genetic Analyzer (Applied Biosystems) or a 3500 Genetic Analyzer (Applied Biosystems). A second DNA extraction and PCR amplification was performed in all positive samples, followed by DNA Sanger sequencing of both strands. All *POLE* variants were described according to the LRG_789 (NM_006231.3) and to the Human Genome Variation Society (HGVS) guidelines.

### Microsatellite instability and MMR immunohistochemistry analysis

Microsatellite instability was performed in the tumors with *POLE* mutations, using a set of five mononucleotide microsatellite markers (BAT26, BAT25, NR21, NR22, and NR24) recommended by the National Cancer Institute as an alternative to the Bethesda panel 13. PCR products of the microsatellite sequences were analyzed for length variations on an ABI PRISM^™^ 310 Genetic Analyzer (Applied Biosystems). Allele sizes were determined using the GeneMapper^®^ software version 3.7 (Applied Biosystems). Tumors were characterized as microsatellite instability‐high (MSI‐H) if they had instability at >30% of *loci*, microsatellite instability‐low (MSI‐L) if unstable at <30% of *loci*, and MSS if they showed no instability at any *loci*. The tumors with *POLE* mutations were also assessed for *MLH1*,* MSH2*,* MSH6*, and *PMS2* immunoexpression performed as previously described 14.

### Oncogene mutation testing

Mutation screening of *KRAS* (LRG_344, NM_004985.4; exons 3 and 4) and *PIK3CA* (LRG_310, NM_006218.2; exons 10 and 21, formerly known as exons 9 and 20) was performed as previously described by our group 15. For *NRAS* mutation analysis (LRG_92, NM_002524.3; exons 2, 3, and 4), we used the same approach as for *KRAS*, using primers designed with Primer‐BLAST (http://www.ncbi.nlm.nih.gov/tools/primer-blast/; primer sequences available upon request).

The *BRAF* c.1799T>A, p.Val600Glu (LRG_299, NM_004333.4) mutation was screened in the tumors with *POLE* mutations by PCR amplification and high‐resolution melting (HRM) analysis on a LightCycler‐480 II Real‐Time System (Roche Applied Science) according to Mancini et al. 16. As a confirmation of this technique, single‐nucleotide primer extension (SNuPE) was performed following the SNaPshot Kit (Applied Biosystems) manufacturer's protocol.

## Results

**Figure 1:**
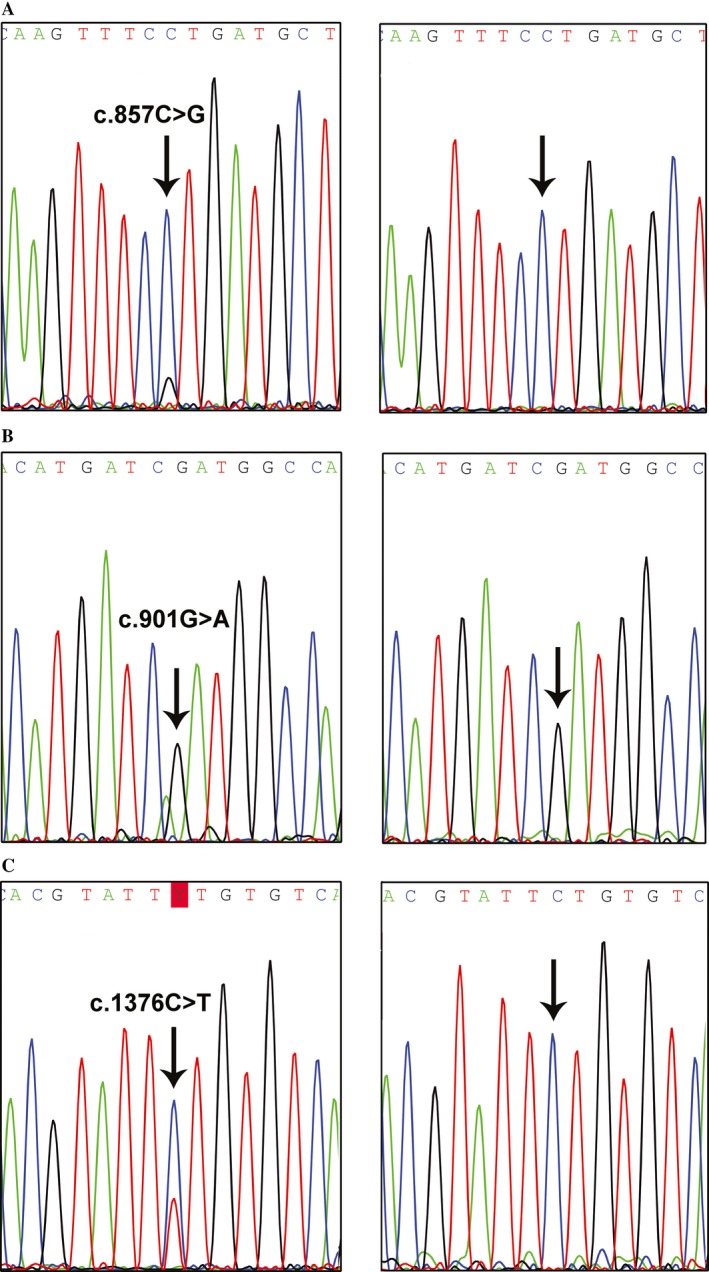
(A) DNA sequence electropherograms obtained from the tumor T286 (left) present the mutation *POLE* c.857C>G, p.Pro286Arg, whereas the adjacent mucosa is normal (right). (B) DNA sequence electropherograms obtained from tumor T368 (left) present the mutation *POLE* c.901G>A, p.Asp301Asn, whereas the adjacent mucosa is normal (right). (C) DNA sequence electropherograms obtained from tumor T368 (left) present the mutation *POLE* c.1376C>T, p.Ser459Phe, whereas the adjacent mucosa is normal (right).

We searched for mutations in the exonuclease domain of the *POLE* gene (exons 9–14) in tumor DNA samples from 307 CRCs. Three heterozygous mutations were identified in two cases (T286 and T368), which corresponds to a frequency of somatic *POLE* mutations of 0.65%. One tumor (T286) presented one *POLE* missense mutation (c.857C>G, p.Pro286Arg) (Fig. 1A), and another tumor (T368) presented two *POLE* missense mutations (c.901G>A, p.Asp301Asn (Fig. 1B); and c.1376C>T, p.Ser459Phe (Fig. 1C)). In order to determine the somatic or germline nature of the mutations found in the tumor tissues, we analyzed DNA samples extracted from normal mucosa adjacent to the tumor, and none of the mutations found in the tumors were present in normal cells of the patients (Fig. 1), indicating that all the mutations found are somatic.

Case T286, with the *POLE* mutation c.857C>G, p.Pro286Arg, was a man who was diagnosed at age 28 years with a moderately differentiated ascending colon adenocarcinoma. Histopathologically, the tumor displayed a cribriform pattern and it was staged as pT4NxMx, according to the TNM classification. The tumor was also tested for mutations in exons 2, 3, and 4 of the *KRAS* and *NRAS* genes, codon 600 of the *BRAF* gene, and exons 10 and 21 of the *PIK3CA* gene, and showed a mutation in *PIK3CA* exon 10, namely the c.1624G>A, p.Glu542Lys (previously reported by our group 15).

Case T368, with the *POLE* mutations c.901G>A, p.Asp301Asn, and c.1376C>T, p.Ser459Phe, was a man who was diagnosed at age 49 years with a mucinous adenocarcinoma located in the cecum and invading the muscle layer. There were also tubular adenomatous polyps with low‐grade dysplasia. The adenocarcinoma was well differentiated and, according to the TNM classification, the tumor stage was pT2N0M0 at the time of diagnosis, but 2 years later, a pelvic recurrence developed. This tumor was also investigated for *KRAS*,* NRAS*,* BRAF*, and *PIK3CA* mutations, and a mutation in *KRAS* exon 4 was found, namely the c.351A>C, p.Lys117Asn.

The two cases with *POLE* mutations were studied by immunohistochemistry, and both showed normal expression of the mismatch repair (MMR) proteins. They were also evaluated for MSI, and the tumor with the *POLE* mutation c.857C>G, p.Pro286Arg, displayed instability only at the BAT25 *locus*, being therefore classified as MSI‐L. The tumor with the two *POLE* mutations, the c.901G>A, p.Asp301Asn, and the c.1376C>T, p.Ser459Phe, did not exhibit any alteration in the five markers, and it was consequently classified as MSS.

## Discussion

In this study, we identified three *POLE* mutations in two of 307 CRCs, a frequency (0.65%) that is lower than some previous reports (3–12.3%) 5, 8 and comparable with that of recent study of Domingo and collaborators (1%) 17. The selection criteria could explain the differences between frequencies of somatic *POLE* EDMs observed. While our cases represent a consecutive series of advanced colorectal tumors not selected for MSI status, the series described by Stenzinger and coworkers, the study with the highest frequency so far described, consisted of CRCs with MSS phenotype, which likely increases the frequency of somatic *POLE* mutations as they have previously been seen mostly in MSS tumors 8. On the other hand, the TCGA cases represent a series of colon or rectum adenocarcinomas of patients undergoing surgical resection, regardless of surgical stage, histologic grade, or MSI status 5, and the series described by Domingo and collaborators consisted in a study population of predominantly stage II and III CRC 17. The three *POLE* EDMs here identified were the c.857C>G, p.Pro286Arg, the c.901G>A p.Asp301Asn, and the c.1376C>T p.Ser459Phe. The p.Pro286Arg and the p.Ser459Phe were previously reported as somatic mutation hotspots 18, whereas the p.Asp301Asn mutation was previously described by Stenzinger and colleagues in only one tumor 8. All these alterations are predicted to have a direct effect on the proofreading function 6, 10. Interestingly, the *POLE* p.Asp301Asn and p.Ser459Phe mutations were found in the same tumor. It was not possible to clarify whether these mutations were present in the same clone (in *cis* or in *trans*) or in different clones, being therefore unknown whether the two mutations occurred simultaneously, whether the presence of one mutation resulted in another mutation in the same clone, or whether these mutations in the same tumor are an example of intratumor heterogeneity. Stenzinger and coworkers also identified six cases with two different *POLE* exonuclease domain mutations 8.

The two tumors harboring the *POLE* EDMs we here report were from patients diagnosed at a younger age (28 and 49 years) compared with the median age at diagnosis in our series (59 years), which is consistent with recent studies 17, 19. Furthermore, the p.Pro286Arg mutation was found in a tumor of a 28‐year‐old man, which is in agreement with two previous studies that demonstrated an association of this particular mutation with early‐onset CRC 20, 21. Additionally, we observed that the tumor T286 was MSI‐L, with just one positive marker, and the tumor T368 was MSS. These data concur with those reported by the TCGA project and by Stenzinger and coworkers, showing that most CRCs with *POLE* EDMs are not MSI‐H 5, 8, 17. According to the literature, these tumors with *POLE* EDMs have a hypermutated phenotype associated with loss of the *POLE* proofreading function and present a high mutational burden in the known CRC driver genes 7, 22. However, these mutations in CRC driver genes are often different from the common hotspots in those genes 7. In our study, we observed that none of the two *POLE*‐positive tumors presented *NRAS* or *BRAF* mutations, but tumor T286 exhibited the p.Glu542Lys *PIK3CA* mutation and tumor T368 displayed the p.Lys117Asn *KRAS* mutation. Both mutations are outside the most common hotspots in those genes, thus confirming earlier observations 7. It is therefore likely that CRCs with *POLE* EDMs define a hypermutated, non‐MSI‐H, group of tumors 5, but it is not yet known whether this disease subset follows one of the previously established CRC pathways or whether they represent a different pathway.

We could not assess whether *POLE* mutations have prognostic or predictive implications due to the small number of mutations found. Nevertheless, Stenzinger and collaborators reported that patients with stage III/IV tumors harboring *POLE* mutations had a statistically significant increased mortality 8. On the other hand, available data on EC suggest that patients with *POLE* EDMs have significantly better outcomes 9, 23, 24. It is unclear how the same pathogenetic mechanism would drive opposite prognosis in the different tumor types, so further studies are necessary to comprehend the biological and clinical impact of these mutations in CRC and in other malignancies. Alterations of the proofreading function may be selectively deleterious to the cell, so it would also be important to determine whether proofreading deficiency has any effect on polymerase processivity. Furthermore, similarly to other examples of targeted therapy based on deficient DNA repair mechanisms 25, 26, it would be important to explore how the hypermutated phenotype associated with *POLE* mutations may be used therapeutically, especially in the context of advanced CRC.

## Conflict of Interest

None declared.
